# Complex Contact Network of Patients at the Beginning of an Epidemic Outbreak: An Analysis Based on 1218 COVID-19 Cases in China

**DOI:** 10.3390/ijerph19020689

**Published:** 2022-01-08

**Authors:** Zhangbo Yang, Jiahao Zhang, Shanxing Gao, Hui Wang

**Affiliations:** 1 School of Humanities and Social Science, Xi’an Jiaotong University, Xi’an 710049, China; yang_zb@xjtu.edu.cn; 2 Institute for Empirical Social Science Research, Xi’an Jiaotong University, Xi’an 710049, China; 3 School of Social Development and Public Policy, Fudan University, Shanghai 200433, China; 4 School of Management, Xi’an Jiaotong University, Xi’an 710049, China; gaozn@xjtu.edu.cn (S.G.); wh_xjtu@stu.xjtu.edu.cn (H.W.)

**Keywords:** COVID-19, social network analysis, contact network, dynamic network evolution

## Abstract

The spread of viruses essentially occurs through the interaction and contact between people, which is closely related to the network of interpersonal relationships. Based on the epidemiological investigations of 1218 COVID-19 cases in eight areas of China, we use text analysis, social network analysis and visualization methods to construct a dynamic contact network of the epidemic. We analyze the corresponding demographic characteristics, network indicators, and structural characteristics of this network. We found that more than 65% of cases are likely to be infected by a strong relationship, and nearly 40% of cases have family members infected at the same time. The overall connectivity of the contact network is low, but there are still some clustered infections. In terms of the degree distribution, most cases’ degrees are concentrated between 0 and 2, which is relatively low, and only a few ones have a higher degree value. The degree distribution also conforms to the power law distribution, indicating the network is a scale-free network. There are 17 cases with a degree greater than 10, and these cluster infections are usually caused by local transmission. The first implication of this research is we find that the COVID-19 spread is closely related to social structures by applying computational sociological methods for infectious disease studies; the second implication is to confirm that text analysis can quickly visualize the spread trajectory at the beginning of an epidemic.

## 1. Introduction

At the end of 2019, an outbreak of COVID-19, a novel coronavirus, rapidly spread in a very short time, becoming the largest “black swan” event of the 21st century. By January 2022, the cumulative number of confirmed COVID-19 cases exceeded 286 million and the death number exceeded 5429 thousand worldwide. The Delta and Omicron variant of the virus cased the new wave of spread across the globe at the end of 2021 [1,2,3]. As a result, the development of vaccines has become an important focus [4,5]. The epidemic has had a huge impact throughout the world, from public health to many aspects of the economy and society, and no country or region could stay away from it. Thus, to understand the evolution, prevention, and control of the epidemic, it is vital to analyze the spatial and temporal co-occurrence of confirmed cases. In addition, using real-world data on disease transmission is crucial during the early stages of an epidemic to ensure the accuracy and efficacy of nonpharmacological interventions. To achieve this goal, the contact network analysis based on real-world transmission data can effectively capture the dynamic transmission process of viruses among individuals and reflect the development of the epidemic through visualization and network modeling [6,7,8,9,10]. The study of contact relationships between patients at the beginning of the outbreak of COVID-19 can also help the prevention of possible future outbreaks.

A large number of researches have gradually improved our understanding of diseases and our ability to fight them. Existing studies on the spread of infectious diseases are mainly based on three methods. The first method is the construction of corresponding models to predict the spread of diseases based on differential equations, such as the classical SI (Susceptible-Infective) [11,12], SIR (Susceptible-Infective-Recovered) [13,14], and SEIR (Susceptible-Exposed-Infective-Recovered) [15,16,17] models. The second method is regression models [18,19] and simulation calculations [20]. The third method is contact network analysis, which is used widely in health departments via contact tracing [21,22,23,24]. Different research methods have deepened our understanding of disease transmission. However, these studies still have limitations. Differential equation models ignore the heterogeneity of individual social interaction patterns. Regression and simulation methods fail to show the microscopic mechanisms of disease transmission. The existing contact network studies lack the support of real-world disease transmission data and fail to show the dynamic evolution of disease spread. To an extent, it reduces the explanatory power of the study on the transmission of diseases [25].

To address these problems, the objective of this study is to build a contact network from real-word cases and calculate related network indicators to reveal the pattern of epidemic transmission at the beginning stage. COVID-19 has a typical “human-to-human” characteristic. Through human-to-human contact and interaction, the epidemic spreads rapidly, resulting in aggregated infections and multiple simultaneous diagnoses. Interpersonal interactions and contacts can reflect the transmission trajectory of the virus, forming a contact network among confirmed cases. The contact network is therefore a duplication of the virus transmission network, which can reflect the disease transmission process at individual level [6]. Meanwhile, the relevant indicators of contact network can be studied by means of social network analysis [25]. In addition, cases published during an outbreak are an important source of real-world data for studying disease outbreak and spread. Therefore, we use case texts to study the disease outbreak and virus transmission pattern.

Specifically, this paper first constructs a dynamic contact network of confirmed cases by mining the texts of case studies during the COVID-19 epidemic outbreak in China. Secondly, based on the network, we calculate the corresponding network indicators and analyze the network structure. The data used in this paper are obtained from the confirmed cases published on the official websites of the Health Commissions of eight areas in China. We chose these regions because detailed information on patient-to-patient contacts is revealed by their case reports.

The main contributions of the study are as follows. First, we confirm that virus transmission is related to the strength of ties in social network, which implies viruses are closely related to human social structures, and that control for diseases is a problem of not only natural science, but also social science. Second, by mining case texts, we combine the contact network analysis method with real-world cases, overcoming the limitations of statistic models and simulation method, presenting the process of disease transmission and spread at the microscopic level. Theoretically and methodologically, this paper enriches the idea of studying the transmission pattern of infectious diseases and emphasizes the need to utilize data from reported cases.

## 2. Theoretical Background

### 2.1. Social Science Research during the Epidemic

The prevention and control of an epidemic outbreak is not only a natural science issue involving virology, epidemiology and immunology, but also a social science issue involving organizational mobilization, social mentality, mental health and economic impact [26,27]. Since the outbreak of COVID-19, relevant issues have been actively explored in the field of social sciences. Many studies have emerged from the fields of public health, sociology, economics and management, which have played a crucial role in the understanding, prevention and control of the pandemic [27].

During the epidemic, physical quarantine and distance-increasing interactions have a great impact on people’s physiology and psychology. Bian et al. constructed the concept of “virus-combat social capital”, which specifically includes the effectiveness of physical isolation, the closeness of relational interactions, and the heterogeneity of information sources [28,29]. In addition, social mindset and mental health issues during quarantine have received much research attention [29,30]. Catastrophic events can reveal some social conditions that exist in daily life, but are often overlooked [31], especially social inequalities. For example, in gender studies, an epidemic may result in higher unemployment rates in industries with more interpersonal contact, where women were overrepresented. The absence of child care institutions also increased the burden of mothers in the family [32]. Therefore, the epidemic has exacerbated the gender inequalities that already existed within families and workplaces [32]. Wu et al. examined the impact of socioeconomic status on health inequalities and found that an individual’s socioeconomic status can influence access to daily protective gear, employment status and community environment, which in turn can affect the risk of infection as well as mental health [33].

Published cases of COVID-19 contain basic information of sex, age, and diagnosis date of confirmed cases, which are an important data source for analyzing the epidemic. A number of studies have retrospectively analyzed the basic characteristics of COVID-19 cases based on published information, including demographic characteristics such as gender, age, etc., as well as clinical diagnostic information such as case’s origin place, onset time and diagnosis time. According to a study of the initial 41 cases of COVID-19, the median age was 49 years old, with 66% cases having a history of exposure to the Wuhan South China Seafood Market [34]. Yang painted a digital portrait of COVID-19 cases in Shaanxi Province of China based on 237 cases and found that these cases were predominantly imported cases in early stages and mostly were middle-aged males [7]. These studies conducted retrospective analyses based on cases using text mining methods, but mostly used descriptive statistics and generally lacked in-depth mining of massive case data.

### 2.2. Social Networks and Disease Transmission

The spread of infectious diseases requires three basic components: infectious source, transmission route, and susceptible population. SIR models and their evolutionary branch models use differential equations to make simulations of disease outbreak and transmission. Furthermore, some other variables are added to expand the explanatory scope of these models. For instance, researchers considered the dissemination of epidemic-related information and constructed a UAU-SIS model, where U and A represent awareness of relevant epidemic information, respectively [35]. In addition, linear regression models [18] as well as autoregressive models [19] have been applied to epidemic-related studies. The adoption of multiple models has enriched the research in this field. However, the above studies lack the support of case data in real-world. Sometimes they only draw static indicator figures such as number of confirmed cases and focus on the prediction or fitting of the infection rate, while failing to show the dynamics of disease transmission from the perspective of microscopic individual interaction process. In this regard, the microscopic spread mechanism of viruses can be analyzed by means of social network analysis.

Viruses can spread along human social ties, forming a contact network among patients [21,22,23]. Contact networks can be used to study the process and pattern of infectious diseases transmission. In such networks, nodes represent patients diagnosed with the disease, and edges between nodes represent the contact relationship between patients, i.e., the transmission path of the virus. Based on the view of the contact network, some researchers have carried out studies on the epidemic pattern of infectious diseases from the perspective of network dynamics, which demonstrate the ability of social network analysis to explain the real world [21,36,37,38]. Researchers have studied the spread of diseases such as the Black Death [23], AIDS [39] and SARS [40] in the human world from the perspective of contact network.

For example, Eubank et al. combined the contact network with simulation to construct a dynamic bipartite graph of individuals and locations to simulate smallpox outbreaks in urban social networks. They found that the individual contact network was a small world network and the location network was a scale-free network, so the authors suggested that targeted vaccination and early outbreaks surveillance could be an alternative to mass vaccination in the control of pandemic [6]. Unlike previous studies, this paper did not use differential equations, but used the contact network method to model the outbreak of virus, thus providing a more microscopic and precise description of the disease. However, the settings about parameters in this study such as the infection rate, fatality rate, and incubation period of smallpox were based on assumptions rather than real-world data of disease transmission, so it may not fully display the process of disease spread in a real outbreak. In addition, researchers emphasized the application of contact network method in the study of the COVID-19 outbreak to better reflect a more realistic population movement [41]. A study modeled the two-stage outbreak of COVID-19 on the Diamond Princess cruise ship and estimated the transmission rate and the basic reproduction number R_0_ [42].

The above studies used different contact network models to simulate the outbreak, but data sources were mostly social media networks and Wi-Fi networks, which generally lack disease transmission data in the real world and could not reflect the contact network among individuals. Therefore, these data limitations reduced the explanatory power of these studies to some extent.

In modern society, where people interact frequently, the structure of social interaction has become a key factor in disease transmission. A small-world type of interpersonal network, Watts and Strogatz proposed, has a dense local connection structure with occasional long-distance connections, connect different clusters together [43]. In this case, the overall contact network of human society is a giant component, in which long-distance edges can form the center of a disease outbreak at two different locations at the same time [6]. In this kind of network, the virus can spread to everyone in the entire population through a small number of interpersonal contacts [22]. This means that the small-world effect allows the viral particles to spread more widely and faster, causing simultaneous oscillations of epidemic beyond the initial outbreak site [44]. Therefore, changes in the structure of social contact networks have an important impact on the development of an epidemic [40]. Contact network structure influences the effectiveness of non-pharmacological intervention strategies and re-open policies [41]. Overall, existing studies confirm that immunization strategies based on network structure, such as degree centrality, clustering coefficient, or modularity, could be more effective [45,46,47].

For the COVID-19 epidemic, some researchers have used real-world case data to conduct relevant studies from the perspective of contact network. Azad and Devi collected data from 30 January to 6 April 2020 in India, visualized the trajectory of confirmed cases from abroad to India, and calculated the corresponding network indicators, whereby the epidemic development in India was classified into four stages [48]. Jo et al. constructed a directed infection network based on data of 3283 cases in the Seoul metropolitan area of Korea from 20 January to 19 July 2020. They calculated indicators such as network out-degree distribution, average path length, and network diameter, pointing out that network structure has an important impact on the transmission processes of COVID-19 and health departments should perform improved investigation and tracking of cases exposure history [49]. These studies, based on real disease infection data, deepen our understanding of epidemic transmission and help us to implement more effective prevention and control policies.

In contrast to figures that only depict the number of people with COVID-19, text of case studies can provide more information about the confirmed cases, including the relationship between different cases and their movement trajectories in addition to basic demographic information, thus reflecting the development and evolution of the epidemic at the individual level. In addition to macro-level prevention and control measures, it is also important to provide guidance and regulation on individual behavior, which requires individual data and information in the spread of the epidemic. Therefore, we investigate the network structure of the COVID-19 transmission network based on confirmed cases.

## 3. Data and Method

### 3.1. Data

The data used in this paper were obtained from cases published on the official websites of the Health Commissions of eight provinces and regions in China, including Gansu, Guizhou, Hainan, Heilongjiang, Inner Mongolia, Shanxi, Tianjin, and Yunnan, for a total of 1218 cases from the time the first case was announced (17 January 2020) to 16 February 2020. These regions were selected because their cases published by Health Commissions included specific movement trajectories and contact relations between cases. This period was the first outbreak of the epidemic on a global scale. The Chinese government took several measures to control the spread of the outbreak, including quarantine, universal nucleic acid testing (NAT), the establishment of cabin hospitals, and medical aids to the infected areas [50,51,52,53]. One of the most significant impacts on the outbreak was the city lock-down implemented in Wuhan, Hubei on 23 January 2020.

The collected information in cases were organized, classified, text-mined and coded in anonymized form. Based on specific text descriptions, the demographic and infection characteristics of COVID-19 cases were counted, including gender, age, household registration, place of symptom onset, source of case, time of arrival, time of symptom onset, time of taking medical measures, and time of definite diagnosis. A dynamic contact network within the eight regions was also constructed based on the relation between cases.

### 3.2. Variables

We first classify the virus spread pathway for each confirmed case. In the perspective of social network analysis, ties can be classified into three categories: strong ties, weak ties and strangers, depending on the frequency, familiarity and emotion intensity of each contact [54,55]. Frequency refers to the daily interaction intensity between contacts. The familiarity between two contacts refers to the degree of mutual understanding between them, while the emotion intensity refers to how close they are to one another [54,55]. It has been confirmed that tie strength can affect virus spread process [56,57,58]. Accordingly, by analyzing published case information, we determine whether a case is infected through a strong tie, a weak tie or a stranger. If one case clearly stated that he or she was diagnosed after close contact with family members, friends, or acquaintances confirmed with COVID-19, the case was considered to have been infected through a strong tie. If one case did not have close contact with other confirmed case, the infection route would be determined by other supporting information such as activity track, household location, type of work, and place of employment. For example, if one case only went out for a walk occasionally and did not have close contact with other people confirmed with COVID-19, the infection route was coded to a stranger contact. Otherwise, the infection was caused by a weak tie. If one case is infected through a certain tie, the infection route is coded 1, otherwise coded 0. We also take family infection into consideration. If one case has a family member diagnosed, this case is coded 1, otherwise coded 0.

Second, the contact relationship between confirmed cases is depicted in daily cases published on the Health Commission website, through which we construct and visualize the contact network between confirmed cases. Some studies have shown that network centrality-based immunization strategies are more effective [46,47]. We calculate the relevant network indicators, which are shown in Table 1. Furthermore, referring to Eubank et al. [6] and Jo et al. [49], we also simulate the effect of quarantine policy by deleting nodes with certain value of degree centrality.

## 4. Results

### 4.1. Description Statistics

In this section, we first present the description statistics about diagnosed cases, including demographic information such as gender, age, infection source place (inside or outside the area), and the likelihood of being infected by each type of ties (strong tie, weak tie or stranger). The results are shown in Table 2 and Table 3.

As can be seen in Table 2 and Table 3, there was no significant difference in the percentage of men and women (50.32% vs. 49.68%). In terms of the origin place of infection, 642 cases were infected within the eight areas, accounting for 57.17% of all cases. In terms of the specific tie of infection with the virus, 64.65% cases had the possibility of being infected by strangers. More than 40% cases had the possibility of being infected by a weak tie such as an unfamiliar colleague. More than 65% cases had the possibility of being infected by a strong tie. Close to 40% cases had family members diagnosed, which indicates a greater proportion of infections occurring within family.

**Figure 1:**
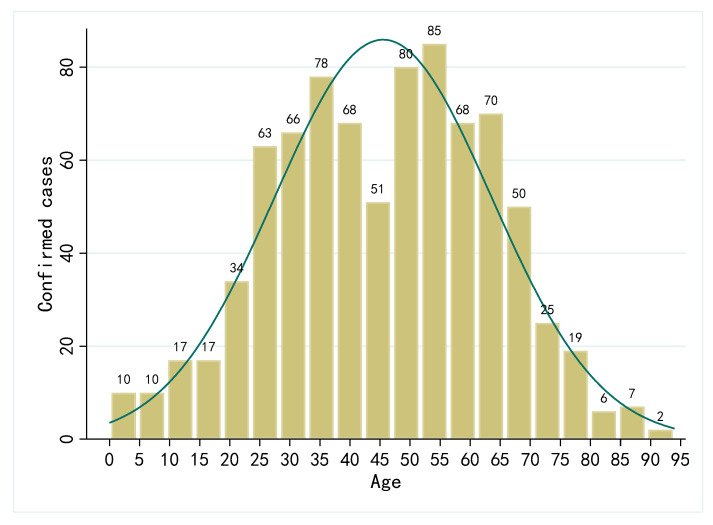
Age distribution of COVID-19 cases in eight regions of China.

The average age of all confirmed cases was 45.51 years old, with the youngest one only 1 month old and the oldest one 94 years old. Figure 1 shows the age distribution of all confirmed cases.

**Figure 2:**
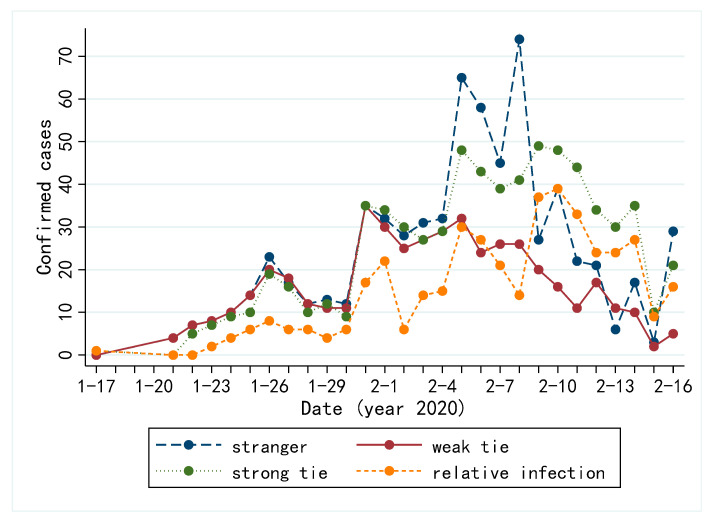
Change of COVID-19 infection by different ties in eight regions of China.

We further analyzed the transmission route of COVID-19 among all confirmed cases. Figure 2 illustrates the trend of infection route over time. In addition to the three social ties, the family member infection route is also depicted. A higher proportion of cases are infected through stranger ties and strong ties.

### 4.2. Contact Networks of COVID-19

Based on the information, we construct dynamic contact networks of confirmed cases in eight areas in China. To present dynamic changes of the network, each network of a certain region is intercepted by typical time intervals for analysis. In the contact network, nodes represent confirmed COVID-19 cases, and edges represent close contact between cases and the presence of a virus transmission route. Each node is numbered, representing its order reported by the local Health Commission. The larger the number is, the later that case was confirmed. Size of node represents the value of its degree centrality. The bigger the size is, the more people got infected directly by that case.

#### 4.2.1. Contact Networks Structure and Visualization

**Figure 3:**
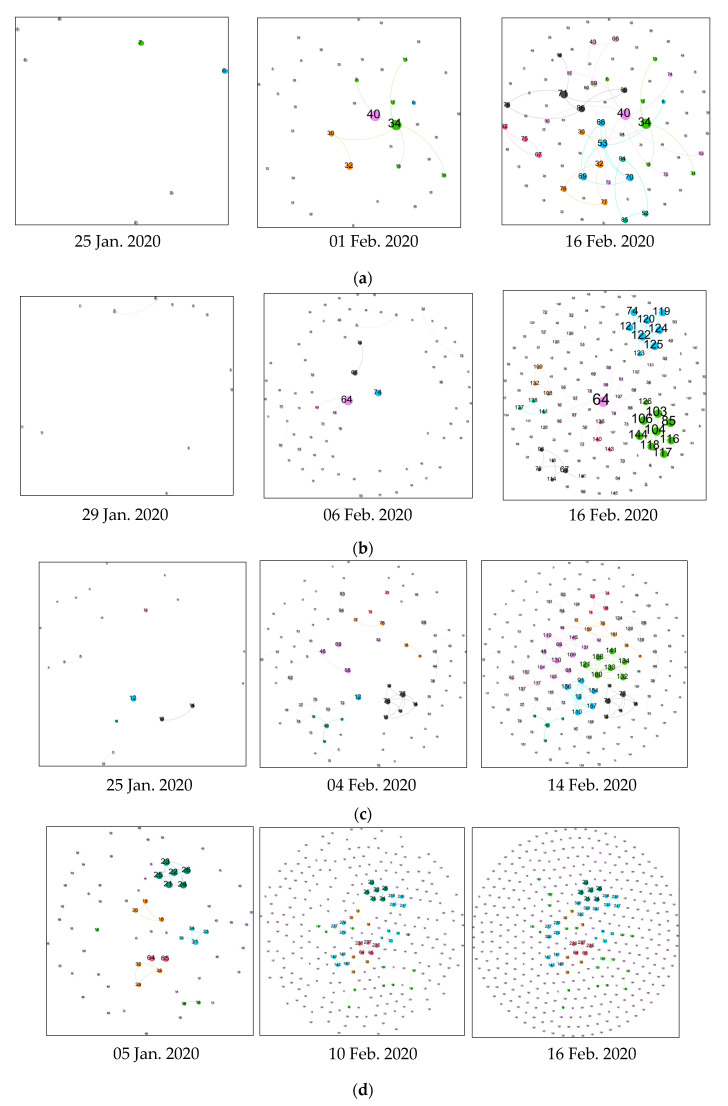

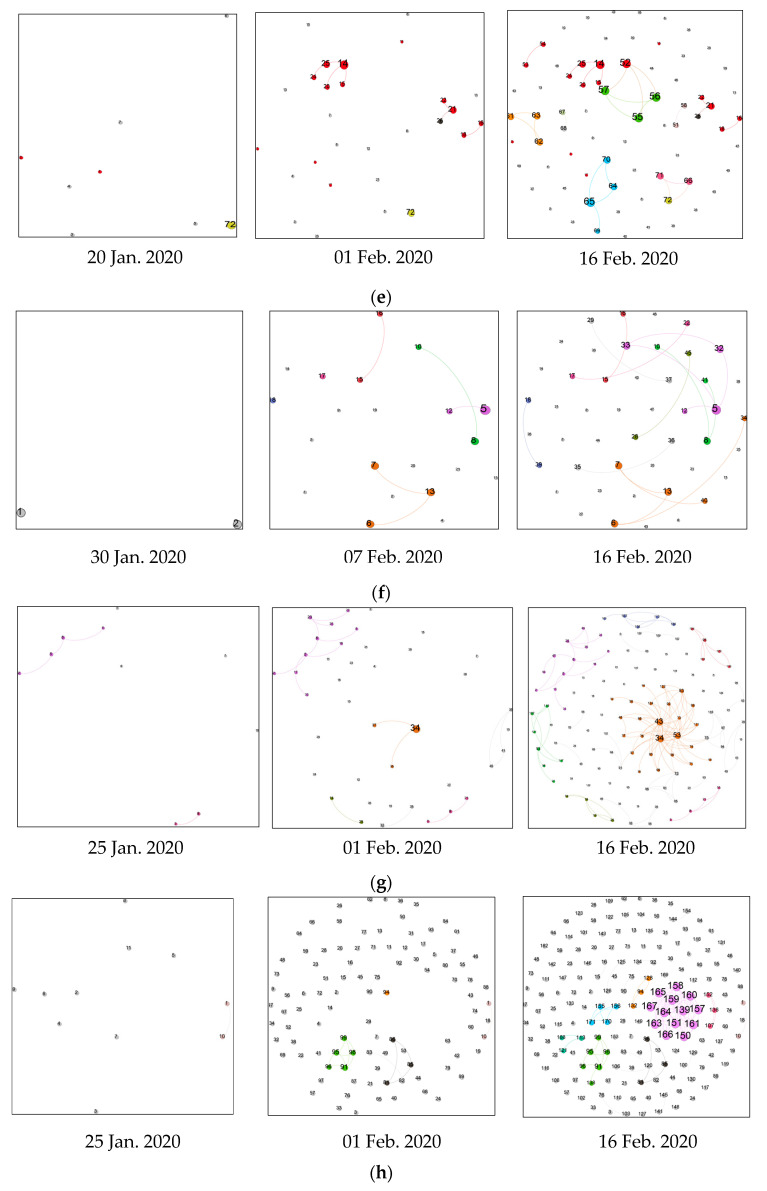
Dynamic contact networks of COVID-19 cases in eight regions of China. (**a**) Dynamic network of Gansu. (**b**) Dynamic network of Guizhou. (**c**) Dynamic network of Hainan. (**d**) Dynamic network of Heilongjiang. (**e**) Dynamic network of Inner Mongolia. (**f**) Dynamic network of Shanxi. (**g**) Dynamic network of Tianjin. (**h**) Dynamic network of Yunnan.

Figure 3 shows the dynamic contact networks in all the eight regions. As can be seen, in the early period, the contact network is sparse, indicating that at early stage the outbreak was dominated by individual infection rather than chain infection. After February 2020, some clusters gradually appeared, which indicates that mass infection emerged over time. In Heilongjiang Province, for example, the first confirmed case was reported on 23 January 2020, and there were only three cases until 31 January 2020. By the middle of the period (5 February 2020), however, the number of confirmed cases in Heilongjiang Province suddenly surged, with 56 new cases confirmed on that day, resulting in a total of 68 cumulative confirmed cases. At the same time, there were several clusters of mass infection. In many provinces, the largest cluster usually included people staying or passing by Wuhan, such as case No. 34 in Gansu Province, case No. 64 in Guizhou Province, case No. 52 in Inner Mongolia, case No. 138 in Yunnan Province, and case No. 26 in Heilongjiang Province.

After 5 February 2020, clusters formed in the mid-term expanded and a few fully connected clusters appeared. Fully connected clusters occurred mostly in families. In a fully connected cluster, family members are infected almost simultaneously, making it difficult to distinguish their transmission pathways and sequences. By the later stage, no more new clusters appeared, indicating that the epidemic was basically controlled. Since various epidemic prevention policies were implemented in different parts of the country, the contact network gradually broke down into many different sub-communities, which indicated that the connectivity of the network was decreasing. In particular, the lock-down of Wuhan also resulted in the absence of new cases in our study areas after 16 February 2020. In addition, there are many isolated nodes in the contact network, some of which might be infected through strangers. Some isolated cases mentioned the source of infection in their case but the clear transmission pathways between them and others could no longer be traced due to insufficient data.

In order to demonstrate the transmission pathways of COVID-19 more clearly, we specifically select some confirmed cases for individual-level analysis. For instance, the largest family infection in Inner Mongolia was a dense cluster formed by cases No. 52, 55, 56, and 57 (Figure 3e). In this cluster, case No. 52 returned from Wuhan and visited his family members, which eventually led to the infection of all close contacts. The largest cluster in Yunnan was formed by cases No. 139, 150, 151, 157, 158, and 160–167 (Figure 3h). In this cluster, case No. 139 who returned from Wuhan participated in a village-wide gathering party, which eventually led to a cluster of infection. As another example, cases No. 64 and 65 (husband and wife) in Heilongjiang Province were diagnosed on 5 February 2020, and they had previously gathered with cases No. 236 and 237 (their sisters) at home of case No. 235 (their mother) on 24 January 2020, so that both their mother and sisters were diagnosed on 9 February 2020 (Figure 3d). This family also formed a relatively large cluster at the later stage. The son of cases No. 64 and 65 returned from Shandong to Heilongjiang on 19 January 2020, arrived at his parents’ home on 22 January 2020, then attended a family dinner on 24 January 2020, and went back to Shandong on 28 January 2020. The son (not be showed in Figure 3) was being treated in hospital because of COVID-19 when his parents were confirmed to be infected. Therefore, it is possible that this family outbreak initially started from the son.

#### 4.2.2. Contact Networks Centrality Analysis

To further analyze the contact network of COVID-19 cases, we calculated relevant network indicators including degree centrality, closeness centrality, betweenness centrality and PageRank scores. The results are shown in Table 4. The mean value of degree centrality was higher than 1 in Guizhou, Hainan, Tianjin and Yunnan, which means a focal case spreads the virus to more than one person on average. The minimum value of degree centrality was 0, while the maximum was 24, indicating that 24 new cases resulted from this case (case No. 34 in Tianjin network). The No. 34 case was an employee of Baodi department store in Tianjin where a mass infection occurred. The largest infection sub-network in Tianjin formed with this case as the center.

The value of closeness centrality represents the proximity of one case to other cases in the contact network. Higher values mean that the epidemic can spread with fewer intermediate patients. Among eight regions, Hainan Province, with rich tourism resources, has the highest mean value of 0.479 for the closeness centrality, which is probably due to the mass infections caused by vacation travel. The lowest mean value of 0.130 for the closeness centrality is in Heilongjiang, which indicates that there are fewer cluster infections in the province. In addition, as can be seen in Figure 3, although some sub-networks formed over time, confirmed cases received timely quarantine and treatment, which prevented further spread of COVID-19.

Betweenness centrality measures the ability of one case to act as a bridge of virus transmission, such as the position of B in an A-B-C triple transmission. Generally, the small value of betweenness centrality indicates less chain transmission during an epidemic in these areas. 

In terms of PageRank scores, the eight regions generally take low value, with the maximum value of only 0.072 and the minimum value of about 0.001. The wide range of PageRank scores indicates that the connectivity of confirmed cases is unevenly distributed in the network.

**Figure 4:**
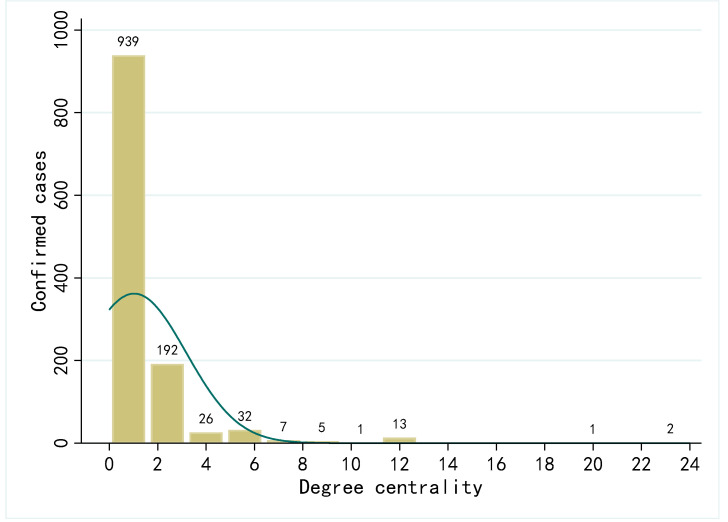
Distribution of degree centrality.

Figure 4 shows the degree centrality distribution of all cases in eight regions. Most cases’ degree centrality is between 0 and 2, and only a few ones have higher values. The overall distribution shows a “long-tailed” characteristic. 

#### 4.2.3. Simulation of Quarantine Policy in Contact Networks

Drawing on the previous researches [6,49], we also run a simulation on the effect of the quarantine policy in epidemic prevention and control. Specifically, by removing the nodes of the certain value of degree centrality in the contact network, we observe the changes of indicators in the overall network to reflect the policy intervention on the spread of COVID-19. The results of the simulation are shown in Table 5. With the deletion of nodes with a high degree, the overall contact network density drops sharply, while the number of components gradually increases. The changes in these two indicators demonstrate that the connectivity of the contact network is declining, and the overall network is gradually becoming fragile. Therefore, when individuals with high transmission risk are isolated, the spread of diseases can be effectively controlled.

## 5. Discussion

In terms of ties strength of the contact network, many infections through stranger tie shows that many cases are infected without contact with colleagues, family members or close friends; many infections through strong tie shows that clustering infections are one of the important causes in the spread of virus. The number of family member infections showed a significant increase in February 2020, probably because many cases had already been infected with COVID-19 during the Chinese New Year but diagnosed in early February 2020 due to the incubation period. During the Chinese New Year, people in China like visiting their family members or taking a vacation. At the same time, the overall trend of all the four types of ties gradually dropped after a small peak in early February 2020, indicating that during the later stage, the epidemic was under control.

Based on the network structure analysis and visualization, we find that the largest clusters of infection in most provinces included cases with a history of residence or travelling in Wuhan, then gathering activities lead to a larger transmission range. Besides, clusters of infection in some areas mostly formed after February 2020, which may be caused by gathering activities during the Spring Festival [59,60,61,62]. This is another proof that control of gathering activities and quarantine of infected populations are especially critical in the prevention and control of the epidemic.

**Figure 5:**
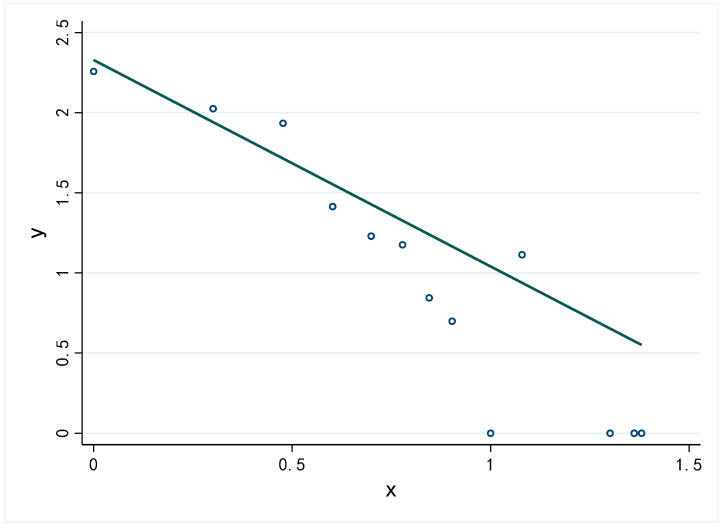
Degree distribution in logarithmic coordinates.

Based on the centrality values in the network, we test whether the degree centrality distribution is in accordance with the power law distribution by fitting the logarithm of degree and frequency [63]. The results are shown in Figure 5. The slope of the fitting line is −1.29 and the goodness of fit (R-Square) is 0.855, which indicates that the degree distribution is consistent with the power law. This implies that the contact network of confirmed cases is a scale-free network, in which only a few nodes have a high degree centrality and most nodes have a low degree centrality. As Meyers et al. pointed out, the spread of disease is particularly not serious in scale-free networks [40]. In addition, there were 17 cases with degrees greater than 10. Given cases with high degree centrality, timely quarantine and medical treatment is key to interrupt further spread of the epidemic.

Similar to the study on the Seoul metropolitan area of South Korea [49] and Shaanxi Province of China [7], the distribution of the patient’s degree in this research is also uneven and follows a power-law distribution. However, in the study on South Korea, a directed transmission network between cases was constructed. Due to data limitations, this article cannot distinguish the infectious directions between confirmed patients, so only an undirected transmission network is constructed. Unlike the research on India [48], we do not construct a regional transmission network based on the travel history of confirmed cases, but this is a good future research direction, which can reflect the network characteristics of virus transmission at the spatial level. Besides, in Shaanxi Province, China, approximately 74% of patients may be infected by strong relationships, while we found that the proportion in other eight parts of China is slightly lower, at approximately 66%. In terms of average age, Shaanxi Province is relatively close to other eight areas of China, with the former being 45.9 years old and the latter being 45.5 years old.

## 6. Conclusions

By collecting and text mining 1218 cases of COVID-19 from eight areas in China at the beginning of the epidemic, this study first illustrates demographical statistics of confirmed cases in terms of gender, age, and source of infection. Then, we classify the types of social ties from which cases are infected. Further, based on the published cases, we construct a dynamic contact network of confirmed cases to demonstrate the trend and transmission of the epidemic. Overall, there are clusters of aggregated infections which formed at first and then gradually expanded, but the growth trend gradually slowed down at the later stage. Meanwhile, the degree centrality of the network showed a power law distribution, with only a few individuals having a high degree centrality, while the overall network was in a low-connectivity state.

The history of humanity’s fight against diseases is also the process of deepening our understanding on related diseases. The differential equation models represented by SI and SIR have produced a large number of findings based on the assumption of homogeneous populations. However, the assumptions of these models are too strict and may ignore the heterogeneity and dynamic interactions within populations. Similarly, few studies based on regression models can reveal the microscopic mechanism of disease transmission [64]. As a consequence, some researchers have proposed to model disease outbreaks from the contact network perspective, such as Newman [21], Eubank et al. [6], and Meyers et al. [40]. The introduction of contact network method, which places more emphasis on the interaction pattern between microscopic individuals, has further improved our ability to study disease transmission patterns. However, it should be noted that contact network simulation models are not based on real-world disease transmission data, so the dynamic perspective may not be taken into consideration, which reduces the explanatory power of them to some extent.

The first contribution of this study is to use text mining and social network analysis methods to construct a dynamic contact network of confirmed cases based on real-world, original case texts. By analyzing and visualizing the contact relationship between cases, we show the development and evolution of the epidemic at the microscopic and individual level and enrich the research ideas in the field of disease transmission. Second, we find that virus transmission is closely related to social network and strength of social ties and is largely dependent on the structure and trajectory of human social activities, which indicates the study of virus transmission requires not only explanation and tracing of natural science, but also a lot of efforts from social scientists.

This research inevitably has some limitations. First, the data format and content published on the website of the Health Commissions vary with areas. For example, contact relationships were not fully reported in some cases. Second, we selected only eight regions of China because reports from these regions are complete. Some other regions only provided the number of confirmed cases without specific information about trajectories of their activity. Therefore, future studies can conduct relevant analyses based on more complete and comprehensive data that can be acquiring from other database sources. Of course, this requires good organization and coordination of epidemiological investigations by public health departments. Furthermore, case information should be uniform and detailed as much as possible to display the true disease transmission process. High-quality epidemiological investigation not only provides first-hand data for disease-related scientific research, but more importantly, provides timely and accurate reference for intervention policy and epidemic control measures. Finally, because the variability of the mutations of the pathology and the heterogeneity of cases due to regions and time is indeed unavoidable, future research can merge contact network studies in different areas and periods to provide a whole picture of the development of COVID-19.

## Figures and Tables

**Table 1:** Contact network indicators.

Indicator	Definition	Equation	Meaning in Contact Network
Degree Centrality	Number of nodes in the network that are directly connected to a focal node	$C_{D}\left(i\right)=\sum_{j=1}^{n}a_{ij}$	Number of contacts with other patients of a focal patient
Closeness Centrality	Proximity of a node to all other nodes in the network	$C_{C}\left(i\right)=\frac{1}{\sum_{j=1}^{n}d_{ij}}$	Proximity of one patient to other patients, with larger values indicating that the epidemic is spreading with fewer intermediate patients and at a faster rate
Betweenness Centrality	The ability of a node to lie on a geodesic path between other pairs of nodes in the network	$C_{B}\left(i\right)=\sum_{j}^{n}\sum_{k}^{n}b_{jk}\left(i\right)$ *j* ≠ *k* ≠ *i*, *j* < *k*	The ability of a patient to act as a bridge in the transmission of the virus, such as the position of B in an A-B-C transmission route
PageRank Scores	The centrality of a node in the whole network rather than ego network by iterative computation	$PR_{i}=\sum_{j\in B_{i}}\frac{PR_{j}}{n_{j}}$	The degree to which a patient is central to the whole contact network
Number of component	A sub-network of a network in which there are paths between any nodes, but there is no any connections between other sub-networks	—	The more components, the sparser the contact network
Density	How closely the network is connected	$D=\frac{2L}{n\left(n-1\right)}$	In a low-density contact network, virus spread becomes difficulty

**Table 2:** Gender and infection source of contact networks in eight regions of China.

Variables	Items	Frequency	Percentage
Gender	Male	546	50.32%
	Female	539	49.68%
Infection source	Inside region	642	57.17%
	Outside region	481	42.83%

**Table 3:** Infection route of contact networks in eight regions of China.

Variables	Items	Frequency	Percentage
Is there a possibility of being infected by a stranger?	Yes	684	64.65%
	No	374	35.35%
Is there a possibility of being infected by weak ties?	Yes	461	43.57%
	No	597	56.43%
Is there a possibility of being infected by strong ties?	Yes	695	65.69%
	No	363	34.31%
Is there a family member being infected?	Yes	418	39.62%
	No	637	60.38%

**Table 4:** Contact network indicators.

Variables	Areas	Mean	S. D.	Min.	Max.
Degree centrality	Gansu	0.867	1.309	0	6
	Guizhou	1.288	2.241	0	10
	Hainan	1.506	1.777	0	6
	Heilongjiang	0.340	0.985	0	5
	Inner Mongolia	0.722	0.996	0	3
	Shanxi	0.681	0.783	0	3
	Tianjin	2.339	3.517	0	24
	Yunnan	1.345	3.187	0	12
Closeness centrality	Gansu	0.304	0.391	0	1
	Guizhou	0.412	0.457	0	1
	Hainan	0.479	0.459	0	1
	Heilongjiang	0.130	0.331	0	1
	Inner Mongolia	0.366	0.452	0	1
	Shanxi	0.426	0.448	0	1
	Tianjin	0.470	0.331	0	1
	Yunnan	0.320	0.459	0	1
Betweenness centrality	Gansu	0.0002	0.001	0	0.0077
	Guizhou	<0.001	<0.001	0	0.0043
	Hainan	<0.001	<0.001	0	0.0022
	Heilongjiang	<0.001	<0.001	0	0.00002
	Inner Mongolia	<0.001	<0.001	0	0.0020
	Shanxi	<0.001	<0.001	0	0.0039
	Tianjin	0.001	0.005	0	0.0385
	Yunnan	<0.001	<0.001	0	0.0003
PageRank	Gansu	0.011	0.012	0.003	0.072
	Guizhou	0.007	0.007	0.002	0.064
	Hainan	0.006	0.004	0.001	0.019
	Heilongjiang	0.002	0.003	0.001	0.014
	Inner Mongolia	0.014	0.012	0.004	0.049
	Shanxi	0.021	0.017	0.005	0.053
	Tianjin	0.008	0.007	0.001	0.046
	Yunnan	0.006	0.006	0.002	0.022

**Table 5:** Results of removing nodes in contact network.

Indicators	Areas	Original Network	Removing Nodes That Degree ≥ 3	Removing Nodes That Degree ≥ 2
**Number of component**	Gansu	60	73	66
	Guizhou	97	106	102
	Hainan	96	98	86
	Heilongjiang	368	363	362
	Inner Mongolia	52	54	52
	Shanxi	32	33	34
	Tianjin	43	61	53
	Yunnan	132	131	128
**Density**	Gansu	0.010	0.003	<0.001
	Guizhou	0.009	0.003	0.002
	Hainan	0.009	0.004	0.002
	Heilongjiang	0.001	<0.001	<0.001
	Inner Mongolia	0.010	0.007	0.003
	Shanxi	0.015	0.013	0.008
	Tianjin	0.019	0.006	0.003
	Yunnan	0.008	0.002	0.001

## Data Availability

We made a video to depict the dynamic change of the contact network in eight areas in China, see https://youtu.be/Cle2A76i2TQ (accessed on 30 November 2021) [65].
